# Multidrug tolerance conferred by loss-of-function mutations in anti-sigma factor RshA of *Mycobacterium abscessus*

**DOI:** 10.1128/aac.01051-24

**Published:** 2024-10-29

**Authors:** Wassihun Wedajo Aragaw, Tewodros T. Gebresilase, Dereje A. Negatu, Véronique Dartois, Thomas Dick

**Affiliations:** 1 Center for Discovery and Innovation, Hackensack Meridian Health, Nutley, New Jersey, USA; 2 Armauer Hansen Research Institute (AHRI), Addis Ababa, Ethiopia; 3 Institute of Biotechnology, Addis Ababa University, Addis Ababa, Ethiopia; 4 Center for Innovative Drug Development and Therapeutic Trials for Africa (CDT-Africa), Addis Ababa University, Addis Ababa, Ethiopia; 5 Department of Medical Sciences, Hackensack Meridian School of Medicine, Nutley, New Jersey, USA; 6 Department of Microbiology and Immunology, Georgetown University, Washington, DC, USA; Bill & Melinda Gates Medical Research Institute, Cambridge, Massachusetts, USA

**Keywords:** drug resistance, tolerance, fluoroquinolone, *Mycobacterium abscessus*, stress response, SigH

## Abstract

Low-level drug resistance in noncanonical pathways can constitute steppingstones toward acquisition of high-level on-target resistance mutations in the clinic. To capture these intermediate steps in *Mycobacterium abscessus* (Mab), we performed classic mutant selection experiments with moxifloxacin at twofold its minimum inhibitory concentration (MIC) on solid medium. We found that low-level resistance emerged reproducibly as loss-of-function mutations in RshA (MAB_3542c), an anti-sigma factor that negatively regulates activity of SigH, which orchestrates a response to oxidative stress in mycobacteria. Since oxidative stress is generated in response to many antibiotics, we went on to show that deletion of *rshA* confers low to moderate resistance—by measure of MIC—to a dozen agents recommended or evaluated for the treatment of Mab pulmonary infections. Interestingly, this moderate resistance was associated with a wide range of drug tolerance, up to 1,000-fold increased survival of a Δ*rshA* Mab mutant upon exposure to several β-lactams and DNA gyrase inhibitors. Consistent with the putative involvement of the SigH regulon, we showed that addition of the transcription inhibitor rifabutin (RBT) abrogated the high-tolerance phenotype of Δ*rshA* to representatives of the β-lactam and DNA gyrase inhibitor classes. In a survey of 10,000 whole Mab genome sequences, we identified several loss-of-function mutations in *rshA* as well as non-synonymous polymorphisms in two cysteine residues critical for interactions with SigH. Thus, the multidrug multiform resistance phenotype we have uncovered may not only constitute a step toward canonical resistance acquisition during treatment but also contribute directly to treatment failure.

## INTRODUCTION

*Mycobacterium abscessus* (Mab) accounts for most pulmonary infections caused by fast-growing non-tuberculous mycobacteria (NTM). There is currently no reliable cure for Mab pulmonary disease (Mab-PD) (1, 2) despite year-long multidrug treatments that include underperforming, parenteral, and poorly tolerated antibiotics (3). Among the factors driving the refractory nature of Mab-PD is intrinsic resistance of the pathogen to many drug classes (4, 5), including antibiotics that are exquisitely efficacious against pulmonary tuberculosis (TB) such as the rifamycins and fluoroquinolones.

The WhiB7-SigH axis has been identified as a determinant of intrinsic multidrug resistance in mycobacteria, through orchestration of an adaptive response to antibiotic-induced and metabolic stresses (6–9). WhiB7 is a master regulator that protects against ribosome-targeting antibiotics and induces transcription of SigH, which itself triggers an oxidative stress response (10). In Streptomyces, Corynebacterium, and mycobacteria, SigH is co-transcribed with and negatively regulated by the anti-sigma factor RshA (11–14).

In a series of genetic, transcriptomics, and biochemical studies focusing on tigecycline resistance, Ngeow and colleagues identified point mutations in *M. abscessus* RshA (MAB_3542c) and SigH, each disrupting interactions between the anti-sigma and sigma factor (14, 15), causing overexpression of SigH (16) and dysregulated stress responses involved in the resistance phenotype (17, 18). In addition, a frameshift mutation in *rshA*, likely causing loss-of-function, similarly conferred tigecycline resistance (16). Consistent with these observations, *sigH* knockout and *rshA* overexpression caused a mild increase in sensitivity (by MIC) to amikacin and tigecycline (19). However, a separate study confirming the role of SigH questioned that of RshA in tigecycline resistance (20), a discrepancy that remains unresolved.

Fluoroquinolones, targeting DNA gyrase, have an excellent track record of safety and efficacy against bacterial infections, including long-term use to treat TB. Moxifloxacin (MXF) is part of the first successful 4-month regimen for drug-susceptible TB (21, 22) and is included in the WHO treatment recommendations against multidrug-resistant TB (23). They are bactericidal to growing bacteria and non-replicating persisters (24, 25), owing to their mechanism of action leading to chromosome fragmentation and reactive oxygen species (ROS) accumulation (26). Although MXF is included in treatment guidelines against macrolide-resistant Mab-PD (3, 27), its clinical utility is questionable due to the wide Mab MIC distributions (28, 29). In two studies where this was tested, the resistance phenotype was not or very rarely (5 out of 114 isolates) attributable to mutations in the GyrA or GyrB subunits of the DNA gyrase (30, 31). This is in sharp contrast with most bacterial infections (32) and suggests that off-target low-level resistance or variable intrinsic resistance of the Mab complex are at play. In Gram-positive and Gram-negative pathogens, indirect resistance mechanisms such as porin loss or efflux upregulation have also been implicated in fluoroquinolone resistance, conferring low-level resistance as a steppingstone toward high-level on-target mutations (33).

Whole genome sequencing (WGS) has seldom been applied to uncover the genetic determinants of fluoroquinolone resistance in Mab clinical isolates. Therefore, the mechanisms of resistance, whether acquired or intrinsic, in isolates carrying wild-type DNA gyrase genes remain unknown. As a first step toward elucidation of the fluoroquinolone resistome in Mab, we selected MXF low- and high-level resistant mutants *in vitro*, identified the resistance mutations, and characterized the impact of these genetic alterations on the growth inhibitory and bactericidal effects of MXF and all major antibiotics used in the treatment of Mab-PD. We found a series of loss-of-function mutations in RshA conferring various patterns of multidrug resistance versus tolerance, consistent within antibiotic classes. The results reveal that very small changes in growth inhibition, likely missed in clinical susceptibility testing, can translate into pronounced drug tolerance *in vitro*.

## RESULTS

### Selection for low-level MXF resistance identifies *rshA* mutants

**Fig 1:**
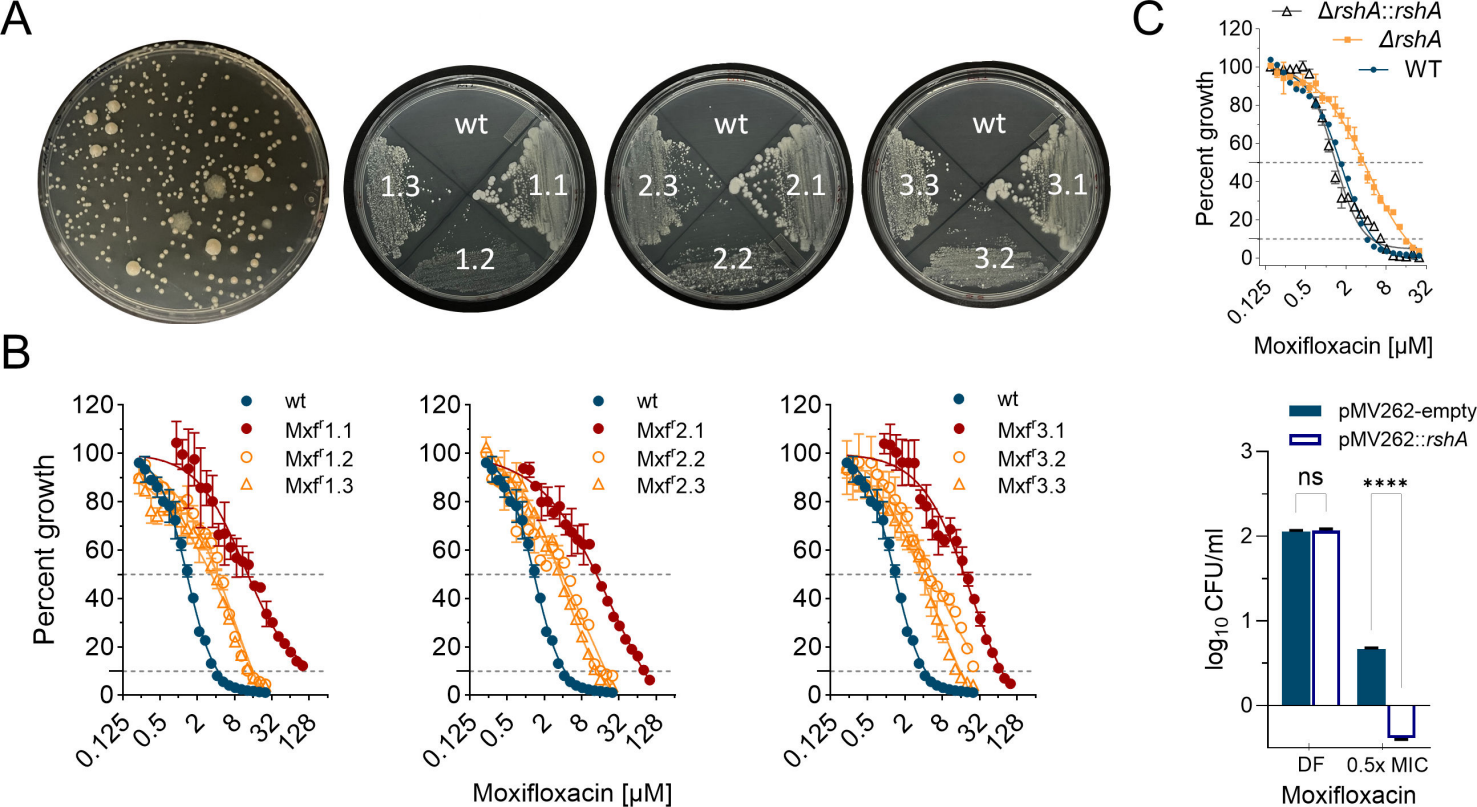
Isolation and characterization of MXF-resistant mutants in *M. abscessus* (Mab) ATCC 19977. (**A**) Diversity of colony morphology on drug-containing agar of mutants selected for MIC and WGS, from three independent experiments. (**B**) Dose-response MIC of wild-type Mab ATCC 19977 and three representative resistant mutants from each experiment. Dark-red curves (high-level resistance) are from missense mutants in *gyrA*; orange curves (low-level resistance) are from frameshift mutants in *rshA* (described in Table 1). (**C**) Recapitulation of MIC shift in a genetically engineered *rshA* knockout, and effect of *rshA* overexpression on MXF’s bactericidal activity at half MIC. DF, drug free.

To identify Mab pathways involved in MXF resistance, we first determined its MIC on 7H10 solid medium and found a significantly higher MIC (100 μM) than in 7H9 liquid medium (~4 μM). Reduced susceptibility of *M. tuberculosis* to fluoroquinolones on solid medium compared with liquid MIC assays has been described previously (34). We then selected spontaneous resistant mutants at 2× the agar MIC in three independent experiments. Colonies with clearly distinct morphologies (Fig. 1A) appeared at different frequencies: small and large smooth colonies emerged at approximate frequencies of 10^−6^ and 10^−8^, respectively. In addition, rough variants were isolated with a low frequency. Representative colonies from each category were retained for dose-response MIC (Fig. 1B) and WGS. We found that all high-frequency/small colonies harbored frameshift mutations in *rshA* leading to loss-of-function, and low frequency/large colonies carried missense mutations in *gyrA*. One of the rough variants carried mutations in *rshA* and MAB_4099c, the latter known to be involved in smooth-to-rough morphotype switching (35) (Table 1).

**TABLE 1:** Genetic analysis of MXF-resistant *M. abscessus* ATCC 19977 mutants

Expt	Strain/mutant ID	MIC_90_ (μM)^*a*^		gyrA/gyrB (aa)	Gene name: polymorphism (mutation type^*b*^)	Other gene(s) with polymorphisms	Colony morphology
		MXF	CLR				
	wt	4	3	wt/wt	wt	wt	
1	Mxf^r^1.1	100	0.8	D96Y/wt	gyrA: G286T/D96Y (ms)		Large smooth
	Mxf^r^1.2	13	3	wt/wt	MAB_3542c: Ins197C (fs)		Small smooth
	Mxf^r^1.3	13	3	wt/wt	MAB_3542c: Ins194_195AAC (non-fs)		Small smooth
2	Mxf^r^2.1	80	0.8	D96N/wt	gyrA: G286A/D96N (ms)		Large smooth
	Mxf^r^2.2	13	3	wt/wt	MAB_3542c: Ins137A (fs)	MAB_4099c: T1265A and G1266A/M422K (ms)	Rough
	Mxf^r^2.3	13	3	wt/wt	MAB_3542c: Ins16C (fs)	MAB_2738c: Ins324C (fs)	Small smooth
3	Mxf^r^3.1	64	0.8	D96G/wt	gyrA: A287G/D96G (ms)		Large smooth
	Mxf^r^3.2	25	12.5	wt/wt	MAB_3542c: Ins5C (fs)	MAB_3302: Ins198C (fs)	Small smooth
	Mxf^r^3.3	13	3	wt/wt	MAB_3542c: Ins123GC (fs)	MAB_3082: G277T/A93S (ms)	Small smooth

^*a*^ MIC_90_, minimum concentration that inhibits 90% growth. Clarithromycin (CLR) was used as assay control.

^*b*^ Ms, missense mutation; fs, frameshift mutation; non-fs, non-frameshift insertion or deletion.

Deletion and overexpression of *rshA* were introduced in Mab ATCC 19977 to confirm the resistance phenotype of the knockout and examine potential hyper susceptibility of the overexpressing strain. We first generated growth curves of the parent, knockout, and complemented strains to rule out fitness differences, which could impact measurements of growth inhibition. The three strains were also streaked out side-by-side on agar. They grew similarly both in liquid and on solid medium (Fig. S1). The *rshA* knockout mutant reproduced the low-level MXF resistance phenotype seen with the spontaneous resistant mutants. RshA overexpression caused mild hyper susceptibility by measure of bactericidal activity (Fig. 1C). These results were consistent with the mild sensitivity phenotype of *rshA* overexpression in a study focusing on the role of SigH in resistance to tigecycline and aminoglycosides (19). Thus, loss-of-function and deletion of *rshA* confer low-level resistance to MXF in the Mab type strain ATCC 19977.

### RshA loss-of-function mutations confer low-level resistance to multiple antibiotic classes

Since *rshA* null mutants were also found to confer low-level resistance to aminoglycosides and tigecycline (18, 19), we measured the MICs of the Δ*rshA* mutant, wild-type, and complemented strains against major antibiotics used to treat Mab-PD or in clinical trials. Additional agents that are not used clinically against Mab-PD were included to examine the impact of Δ*rshA* on common antimycobacterial mechanisms of action. Quabodepistat was added as a strongly bactericidal cell wall inhibitor (36) in clinical trials for TB and trimetrexate as a potent but bacteriostatic Mab DHFR inhibitor (37), approved for the treatment of fungal infections. We found different levels of resistance to different antibiotic classes, generally consistent within classes, as follows: threefold-to-fivefold MIC increase against β-lactams and DNA gyrase inhibitors (MXF and the non-fluoroquinolone gepotidacin), twofold MIC increase against translation inhibitors except omadacycline, and quabodepistat, a DprE1 inhibitor. No or negligible MIC shifts were observed for bedaquiline, clofazimine, trimetrexate, and RBT (Table 2).

**TABLE 2:** Susceptibility of *M. abscessus* ATCC 19977 wild-type, Δ*rshA*, and complemented strains to clinically used antibiotics and other anti-*M*. *abscessus* agents

Drug	Wild type	Δ*rshA*	Δ*rshA::rshA*
	MIC_90_ (μM)^*a*^		
Imipenem	26	200	50
Tebipenem	30	150	43
MXF	4	15	6.5
Fobrepodacin^*b*^	1.6	5.6	2.4
Gepotidacin	29	100	25
Amikacin	12.5	30	7
Tigecycline	2.7	6	1.3
Omadacycline	19.5	21	14
Azithromycin	25	50	25
Clarithromycin^*c*^	3.3	6	3.6
Linezolid	30	50	45
Tedizolid	9	15	9
Quabodepistat^*d*^	8	16	9
Bedaquiline	0.5	0.7	0.4
Clofazimine	12.5	15	10
Trimetrexate	12.5	15	7
RBT	3.1	2.7	3

^*a*^ MIC_90_, minimum concentrations that inhibit 90% of growth.

^*b*^ Formerly SPR720, the prodrug of SPR719.

^*c*^ Measured on day 3 to minimize *erm41* induction.

^*d*^ Formerly OPC-167832.

### RshA deletion confers varying patterns of multidrug tolerance

**Fig 2:**
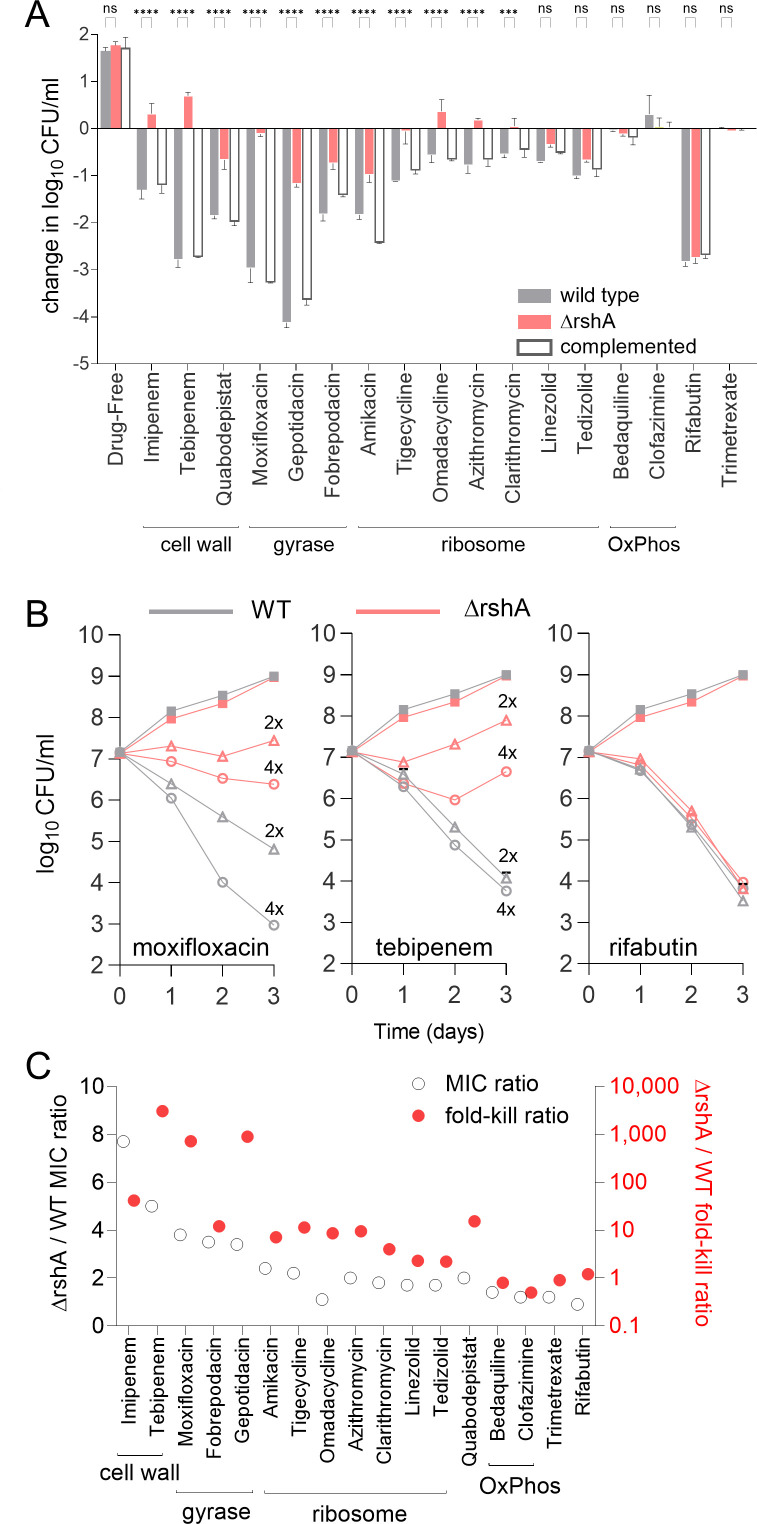
Patterns of multidrug resistance and tolerance in a Δ*rshA* background. (**A**) Effect of *rshA* knockout on the bactericidal activity of anti-Mab agents at approximately 4× their respective MIC after 3 days of incubation. Experiments were carried out twice independently, and one representative set is shown. Data were analyzed using ordinary one-way ANOVA with Šídák’s multiple comparisons test for selected pairs of means as indicated. ns, not significant; ****P < 0.0001. (**B**) Time- and concentration-kill curves for MXF, TBP, and RBT against wild-type (gray lines) and Δ*rshA* (red lines) ATCC 19977. Exponentially growing cultures were treated with 2× or 4× MIC as indicated, and CFUs were enumerated after 1, 2, and 3 days on 7H10 containing 0.4% activated charcoal. (**C**) Comparative impact of *rshA* deletion on growth inhibition (left Y axis, white circles) and killing activity (right Y axis, red circles) of anti-Mab agents described in **A**.

Growth inhibition assays capture MIC-shifted resistance but are blind to drug tolerance, the increased survival under antibiotic pressure (8). To quantify the effect of low-level MIC increases on bacterial survival, we measured the effect of *rshA* deletion on the bactericidal activity of all agents against Mab and generally found a much more profound impact on the killing activity than on the growth inhibitory activity of most antibiotics. This was most pronounced for the β-lactams, DNA gyrase inhibitors, and quabodepistat. For instance, the *rshA* knockout was fivefold less susceptible to tebipenem (TBP) by measure of growth inhibition but exhibited 3,000-fold higher survival compared with the wild type in dose-response bactericidal assays. The weaker bactericidal activity of ribosome targeting agents was also more affected by the *rshA* deletion than their growth inhibitory activity, although the difference was less pronounced. Bedaquiline, clofazimine, and trimetrexate lacked bactericidal activity against both wild-type and Δ*rshA* strains. Remarkably, the strong bactericidal activity of RBT was unaffected by the *rshA* deletion (Fig. 2A). Growth inhibition patterns were confirmed on solid medium for three representative agents with the strongest and contrasting phenotypes: MXF, TBP, and RBT (Fig. S2). We also generated time- and concentration-kill curves for these three drugs against the wild-type and Δ*rshA* strains, confirming the strong attenuation of MXF and TBP bactericidal activity in the *rshA* knockout and the complete lack of effect on RBT’s killing activity (Fig. 2B). Collectively, these results reveal a generally heightened survival and drug tolerance conferred by the loss of *rshA*, to a greater extent than would have been expected based on conventional drug susceptibility testing. The differential patterns of tolerance relative to resistance were conserved within drug classes (Fig. 2C), with the loss of *rshA* inducing the most pronounced tolerance to β-lactams and DNA gyrase inhibitors.

### RBT suppresses drug tolerance conferred by Δ*rshA*

**Fig 3:**
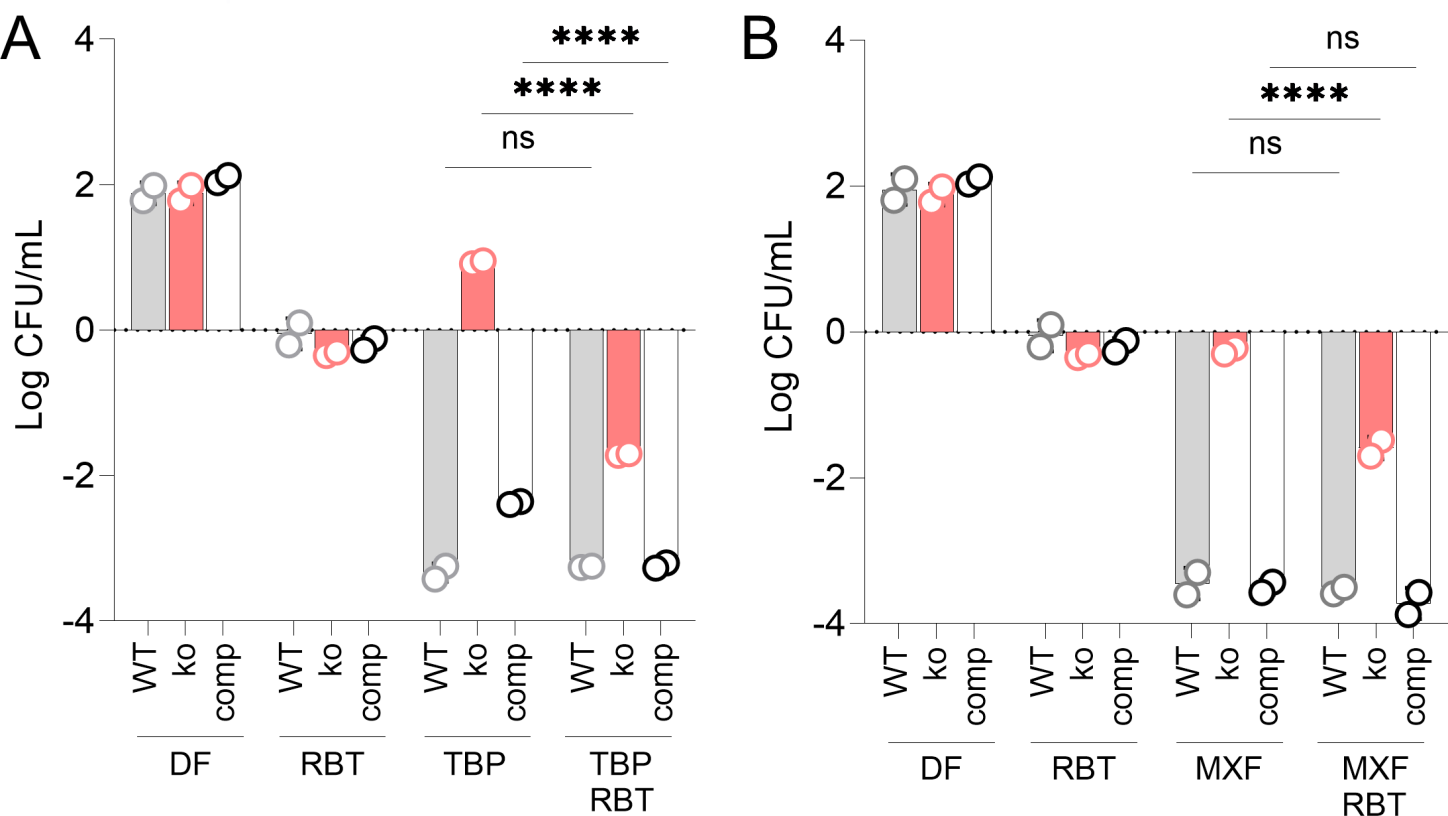
RBT suppresses TBP and MXF tolerance conferred by Δ*rshA* in Mab ATCC 19977. RBT at 0.25× MIC (0.8 μM) was added to 4× MIC of (**A**) TBP (120 μM) or (**B**) MXF (12.5 μM) as indicated. CFU were quantified after 3 days of incubation. ko, *rshA* knockout; comp, complemented strain; DF, drug free. The data were analyzed by two-way ANOVA with Tukey’s multiple comparison test to detect statistically significant differences when RBT was added to TBP or MXF in the wild-type (WT), knockout, or complemented strains. ns, not significant; ****P < 0.0001.

The anti-sigma factor RshA interacts post-translationally with SigH to modulate its activity in response to heat and redox stress (13, 17) and to prevent transcription of SigH-dependent promoters, which becomes constitutive in the absence of RshA. As a transcription inhibitor, RBT may interfere with SigH-dependent transcription of the stress regulon, thereby dampening or even suppressing the increased drug tolerance seen in the *rshA* knockout. To test this hypothesis, we measured the survival of the wild-type and Δ*rshA* Mab strains following treatment with TBP or MXF, alone and combined with RBT at sublethal concentrations. As suspected, RBT partially abrogated the drug tolerance induced by Δ*rshA*, for both TBP and MXF (Fig. 3).

### RshA polymorphisms are present in clinical isolates

To assess the clinical relevance of our multiform resistance findings, we conducted a comprehensive bioinformatics analysis of 10,000 available Mab genomes from the NCBI SRA, a public sequence data repository, to identify frameshift and non-synonymous mutations within the *rshA* gene. We identified three distinct loss-of-function mutations across seven clinical isolates (0.07%) and two missense mutations in Cys residues critical for the interaction between RshA and SigH in three other clinical isolates (0.03%) (Data Set S1).

## DISCUSSION

In this systematic survey of all major antibiotics used in the treatment of Mab-PD, we have shown that *rshA* null mutations confer low to moderate resistance—by measure of MIC increase—to most agents and that this is associated with a wide range of drug tolerance, up to 1,000-fold increased survival of Mab upon exposure to β-lactams and gyrase inhibitors. The mild increase in growth inhibitory activity is consistent with a series of thorough studies by Ng and Ngeow focusing on tigecycline resistance (18) as well as a work on ribosome targeting agents showing mild hyper-susceptibility of a SigH knockout to amikacin and tigecycline (19). Seemingly conflicting results have indicated that Δ*rshA* has a negligible impact on tigecycline bactericidal activity, which the authors attribute to *rshA* knockout having a minimal effect on SigH expression (1.2-fold) under the conditions tested (20). Here, we performed mutant selection, deletion of *rshA*, and complementation confirmed by Sanger sequencing, providing strong confidence in the phenotypes. The discrepancy may be due to differences in the gene knockout strategies.

We found that low-level MIC-shifted resistance conferred by a *rshA* deletion was associated with markedly increased survival for several drug classes. This phenomenon has been observed in *M. tuberculosis* and was coined “multiform antimicrobial resistance” (8). In this elegant study, the authors devised a screening strategy to isolate high-survival mutants without an increase in MIC and found various patterns of heightened survival, persistence, and MIC-shifted resistance in response to induction of WhiB7—an inducer of SigH in Mab (19)—for different antibiotics. Here, we observed different patterns of increased drug tolerance versus MIC increase in Δ*rshA* compared with the wild type, which generally clustered within drug classes. Heightened survival and most pronounced tolerance were observed for the β-lactams and the DNA gyrase inhibitors MXF and gepotidacin, a non-fluoroquinolone gyrase poison (38). Both drug classes inactivate their primary target by generating covalent adducts (β-lactams form covalent drug-target adducts and the two gyrase inhibitors trigger the formation of covalent target-DNA complexes), permanently corrupting their target (26, 39), generating reactive metabolic byproduct accumulation and pronounced antibiotic-induced redox stress as follow-on events (40–42). This may result in stronger induction of the SigH regulon by these drugs than other anti-Mab agents, hence the more profound drug tolerance and survival in the absence of its negative regulator RshA.

Interestingly, the strong bactericidal activity of RBT was unaffected by the loss of RshA, and RBT added to TBP or MXF suppressed the enhanced tolerance of a Δ*rshA* mutant to these two drugs. In a recent study focusing on the impact of WhiB7-mediated metabolic stress on multidrug tolerance, Mab showed decreased susceptibility to cefoxitin associated with tolerance and not MIC-shifted resistance, as the WT and the mutant strains had identical MIC values for this and other antibiotics but achieved increased survival of a persister fraction during the stationary phase only. Cross-tolerance to imipenem and MXF was observed under conditions of enhanced WhiB7 adaptive response, but not to RBT, as seen for our *rshA* mutant (43). It is possible that RBT, in its transcription inhibitor capacity, prevents induction of *whiB7* and *sigH* expression and therefore counters the stress response they trigger. A similar mechanism has been implicated in the synergy between clarithromycin (CLR) and RBT against *erm41*-positive Mab strains, encoding macrolide-inducible resistance (44). In *M. tuberculosis*, antibiotic-induced expression of stress response genes, via activation of isocitrate lyases, conferred tolerance to drugs with various mechanisms of action including rifampicin, but unlike the other drugs, rifampicin tolerance did not involve transcriptional reprogramming (45). While studies in *Escherichia coli* have shown that the bactericidal effect of rifampicin does not entail increased cellular respiration and the accumulation of toxic ROS (46–48), rifampicin does induce hydroxyl radical formation in *M. tuberculosis* (49) and Mab (manuscript in preparation). There might exist a window of concentrations within which rifamycins induce ROS production without sufficiently inhibiting transcription to prevent the SigH-mediated stress response.

Collectively, these studies and the present work describe a common phenomenon by which acquired resistance mutations in genes that appear unrelated to the drug’s primary mechanism of action exploit and enhance an intrinsic pathway of drug tolerance, i.e., the SigH stress response, to provide bacteria with the means to better survive multidrug treatment (6). Mab may be exquisitely prone to the development of resistance and tolerance through this “hitchhiking” strategy given the abundance of intrinsic resistance and tolerance mechanisms it harbors (4, 5, 50).

The presence of *rshA* polymorphisms in several Mab isolates, including non-synonymous mutations in Cys residues critical for interactions with SigH, indicates that this phenomenon may be clinically relevant. Tolerance as a steppingstone that promotes establishment of resistance mutations (6, 51) is thought to occur in two ways: (i) by supporting the continued survival of the population, it extends the window of opportunity for rarer mutations, and (ii) by increasing the number of survivors, it lowers the probability of resistance mutations getting lost during antibiotic exposure (52). In *M. tuberculosis*, functional genetic diversity analyses have revealed various loci associated with drug phenotypes such as low-level MIC increase and tolerance, which predict the development of resistance and treatment failure (53, 54).

The multidrug multiform resistance we describe here, where minor MIC creep leads to profound drug tolerance, is likely to be missed in standard clinical susceptibility assays, which focus on MIC increases that exceed established susceptibility breakpoints. Heightened drug tolerance of bacterial subpopulations can create spatial and temporal pockets of survivors that form the source of disease relapse. Our findings combined with the presence of *rshA* loss of function mutations in clinical Mab isolates suggest it may constitute a step toward canonical resistance acquisition and contribute to treatment failure.

## MATERIALS AND METHODS

### Bacterial strains, media, and culture conditions

*M. abscessus* subsp. abscessus ATCC 19977 was purchased from the American Type Culture Collection. *M. abscessus* strains were grown in Middlebrook 7H9 broth (BD Difco) supplemented with 0.05% (vol/vol) Tween 80 (Sigma), 0.2% (vol/vol) glycerol (Fisher Scientific), and 10% (vol/vol) Middlebrook albumin-dextrose-catalase (BD Difco) at 37°C with agitation at 90 rpm (INFORS HT Multitron Pro). For determination of CFU, bacterial cultures were spread onto Middlebrook 7H10 agar (BD Difco) supplemented with 10% (vol/vol) Middlebrook oleic acid-albumin-dextrose-catalase and 0.2% glycerol and grown at 37°C. When appropriate, agar was supplemented with MXF for the isolation of resistant mutants or 50 µg/mL apramycin or 25 µg/mL zeocin for the selection of transformed bacteria in the generation of recombinant *M. abscessus* strains. *E. coli* strain TOP10 was used for propagation of plasmids and was cultured in Luria-Bertani (LB) broth or on LB agar (BD Difco).

### Drugs and chemicals

MXF, TBP, tedizolid, bedaquiline, omadacycline, RBT, fobrepodacin (SPR720), quabodepistat (OPC-167832), gepotidacin, and trimetrexate were purchased from MedChem Express LLC. CLR, azithromycin, imipenem, amikacin, tigecycline, linezolid. and clofazimine were purchased from Sigma-Aldrich Inc. All chemicals were dissolved in dimethyl sulfoxide (Sigma-Aldrich Inc.) except amikacin and imipenem, which were dissolved in deionized water and sterilized using 0.2-µm Minisart high-flow syringe filters (Sartorius).

### Selection and analysis of spontaneous resistant mutants

Mutant selection was carried out as previously described (55). In brief, bacterial inocula (5 × 10^8^) from mid-log cultures of *M. abscessus* ATCC 19977 were spread on 7H10 agar containing MXF at 2× the agar MIC (100 µM), the lowest concentration that suppressed the growth of wild-type colonies. To confirm resistance, colonies were picked and re-streaked on agar plates containing the same MXF concentration used to isolate the putative mutants. Single colonies from these re-streak plates were then picked, inoculated into 7H9 broth, grown to mid-log phase, and stored with 10% glycerol at −80°C until further analysis.

### Growth inhibition assays

Dose-response growth inhibition was measured using the broth microdilution assay as described previously (55). For each drug, a dilution series starting at the desired highest concentration was dispensed onto flat-bottom 96-well plates using a D300e Digital Dispenser (Tecan). Exponentially growing cultures (optical density or OD_600 nm_ = 0.4 to 0.6) were diluted to a final OD_600_ of 0.05, and 200 µL of these cultures was dispensed to each drug-containing well. Untreated control wells were included on each plate. Culture plates were sealed with parafilm, stored in boxes with wet paper towels, and incubated for 3 days with shaking (90 rpm, Infors HT Multitron). Growth was monitored by measuring OD_600 nm_ using a Tecan Infinite 200 Pro microplate reader (Tecan). Day 0 values were subtracted from the corresponding end-point values. The percentage growth was calculated by dividing the OD _600 nm_ of the drug-containing well by the average OD _600 nm_ of the untreated control wells and multiplying by 100. Dose-response curves were generated by plotting drug concentrations versus percentage growth using GraphPad Prism. Agar MIC, the lowest drug concentration that inhibits the emergence of colonies when plating 10^4^ CFU, was determined by the agar dilution method in accordance with the CLSI protocol (56). A dilution series of the tested drugs was prepared, thoroughly mixed with molten 7H10 agar (~50°C) in 50-mL tubes, and poured into sterile petri dishes. Inocula were prepared by diluting exponential phase pre-cultures to give 10^4^ CFU per spot on the agar. Plates were incubated for 7 (TBP and RBT plates) or 10 (MXF plates) days before pictures were taken. The MIC is defined as the lowest concentration of an antimicrobial agent that fully prevents visible growth, as observed with the naked eye.

### Time-concentration kill assay

Bactericidal activity was determined as described previously (57). Cultures treated with each tested drug at approximately 2× and 4× MIC of MXF (6.2 µM and 12.5 µM), TBP (60 µM and 120 µM), and RBT (6.2 µM and 12.5 µM) were grown as described in the broth microdilution assay for growth inhibition determinations. At 1, 2, and 3 days, cultures were resuspended by pipetting, 20 µL of the cultures was transferred to round-bottom 96-well plates containing 180 µL of 7H9 media for 10-fold serial dilutions, and 10 µL of these cultures was plated at different dilutions. To prevent compound carryover effects, we plated out samples onto 7H10 agar supplemented with 0.4% activated charcoal (C9154; Sigma-Aldrich, USA) for all dilutions. CFU were enumerated after 3 days of incubation, and the reduction in CFU was calculated by comparing CFU counts of the treated cultures to that of the untreated control at time zero. For the RBT stress response suppression experiment, 0.25× RBT MIC (0.8 µM) was selected to prevent growth inhibition or bactericidal effect and was combined with approximately 4× MIC of MXF (12.5 µM) or TBP (120 µM). The cultures were treated for 3 days, and CFU readouts were obtained as described above. Data were analyzed using ordinary one-way ANOVA with Šídák’s multiple comparisons test using GraphPad Prism.

### Whole-genome and Sanger sequencing

Genomic DNA was extracted using the phenol-chloroform method as described previously (58). Targeted sequencing of *gyrA* (MAB_0019) and *gyrB* (MAB_0009) genes was performed by Genewiz Inc. (South Plainfield, NJ, USA) using custom-designed primers listed in Table S1. Whole genome resequencing was carried out to identify additional mechanisms that confer MXF resistance (Novogene Corporation Inc., Sacramento, CA, USA) as described previously (55). The GenBank accession number for the sequence of the parent strain *M. abscessus* ATCC 19977 is CU458896.1.

### Bioinformatics analysis (quality control, alignment, variant calling, and annotation)

Sequence metadata were retrieved from the SRA database using the SRA (Sequence Read Archive) toolkit (version 3.1.0), with the search terms “*Mycobacteroides abscessus*,” and only those entries with a WGS strategy were retained. A total of 9,865 SRA files met these criteria, and the corresponding FASTQ files were downloaded. Quality control and trimming were performed using fastp (version 0.23.4) with default parameters (59). Alignment to the reference genome (GenBank accession number: CU458896.1) was conducted using bwa-mem2 (version 2.2.1) (60) with a minimum mapping quality threshold of 60. The aligned SAM files were converted to BAM, fixed for mate information, sorted, and indexed using samtools (version 1.11) (61), and duplicates were marked using samtools markdup. Variant calling was performed using bcftools (version 1.20) (61) in the multiallelic caller mode with a minimum base quality of 20, a minimum coverage of 10, and a minimum proportion of reads differing from the reference of 0.9. Variants were called using bcftools mpileup and “bcftools call,” followed by filtering with “bcftools filter” based on the specified criteria. Variant annotation was carried out using SnpEff (version 5.2) (62). Following this, SnpSift (version 5.2) (62) was employed to filter variants within the MAB_3542c gene and extract specific fields from the VCF files. Finally, a custom Python script was used to integrate selected fields from the annotated VCF files with key fields from the SRA metadata.

### Generation of MAB_3542c (*rshA*) deletion *M. abscessus* ATCC 19977 mutant

The *rshA* knockout strain was constructed by targeted gene replacement with a linear DNA allelic exchange substrate (AES) using recombineering technique as described previously (63). To construct the AES, DNA fragments containing the upstream (1,000 bp) and downstream (1,000 bp) flanking regions of *rshA* (excluding the four nucleotides overlapping *sigH*) were amplified by PCR using custom-designed primers (Table S1) and cloned into pYUB854-aprR to flank the apramycin resistance cassette. Cloning was verified by Sanger sequencing, and the plasmid backbone was removed by PCR amplification to generate the linear dsDNA AES (see Table S1 for primer list). To generate the recombineering strain, *M. abscessus* ATCC 19977 was transformed with pJV53-zeoR, the plasmid expressing the recombinase enzyme under the control of the acetamide-inducible promoter (pJV53 is a gift from Anil Singh, Northeastern Regional Institute of Science and Technology, Nirjuli, India). Mid-log phase (OD _600 nm_ = 0.4 to 0.6) *M. abscessus* ATCC 19977 (pJV53-zeoR) culture was induced by 0.2% acetamide, incubating for 3 hours at 37°C. To prepare electrocompetent cells, *M. abscessus* (pJV53-zeoR) cultures were washed 3× with cold 10% glycerol and resuspended with 1/100 vol of 10% glycerol. An aliquot of 200 µL culture was transformed with 200 ng AES in a 0.2-cm cuvette by electroporation (Bio-Rad Gene Pulser, setting: 2500 mV, 1,000 Ω, 25 µF) and plated on 7H10 agar containing 50 µg/mL apramycin. Mutant genotypes were confirmed by Sanger sequencing (see Table S1 for primer list). For complementation, we cloned wild-type *rshA* in pMV262 (zeoR) plasmid to express under the constitutive hsp60 promoter. pMV262 (zeoR) was generated by replacing the kanamycin resistance cassette of pMV262 (kanR) (64) with a zeocin resistance cassette under the control of the EM7 promoter. Apramycin was purchased from Alfa Aesar and used at 50 µg/mL. Zeocin was purchased from Thermo Fisher Scientific and used at 25 µg/mL.

## SUPPLEMENTAL MATERIAL

Data Set S1 aac.01051-24-s0001.xlsx Detection of *rshA* polymorphisms among 10,000 genomes publicly available.

Supplemental material aac.01051-24-s0002.docx Table S1; Figures S1 and S2.

ASM does not own the copyrights to Supplemental Material that may be linked to, or accessed through, an article. The authors have granted ASM a non-exclusive, world-wide license to publish the Supplemental Material files. Please contact the corresponding author directly for reuse.
