# Supplementary material for: Multidrug tolerance conferred by loss-of-function mutations in anti-sigma factor RshA of Mycobacterium abscessus
Source: Antimicrob Agents Chemother. 2024 Oct 29;68(12):e01051-24. doi: 10.1128/aac.01051-24 (PMC11619451; doi:10.1128/aac.01051-24)
Supplement: Supplemental material — Table S1; Figures S1 and S2. [file aac.01051-24-s0002.docx]

Multidrug tolerance conferred by loss-of-function mutations in anti-sigma factor RshA in *Mycobacterium abscessus*

Wassihun Wedajo Aragaw ^a^, Tewodros T. Gebresilase ^b, c^, Dereje A. Negatu ^a, d^, Véronique Dartois ^a, e, #^ and Thomas Dick ^a, e, f^

^a^ Center for Discovery and Innovation, Hackensack Meridian Health, Nutley, New Jersey, USA

^b^ Armauer Hansen Research Institute (AHRI), Addis Ababa, Ethiopia

^c^ Institute of Biotechnology, Addis Ababa University, Addis Ababa, Ethiopia

^d^ Center for Innovative Drug Development and Therapeutic Trials for Africa (CDT-Africa), Addis Ababa University, Addis Ababa, Ethiopia

^e^ Department of Medical Sciences, Hackensack Meridian School of Medicine, Nutley, New Jersey, USA

^f^ Department of Microbiology and Immunology, Georgetown University, Washington, DC, USA

Running Title: role of anti-sigma factor in drug tolerance

Keywords: drug resistance, tolerance, fluoroquinolone, *Mycobacterium abscessus*, stress response, SigH

^#^ Corresponding author: [veronique.dartois@hmh-cdi.org](mailto:veronique.dartois@hmh-cdi.org)

| **Plasmid name** | **Properties** | **Reference** |
| --- | --- | --- |
| pJV53 | Acetamidase promoter, Che9c genes 60 and 61, zeo^R^ | Kind gift from Dr Anil Singh, Northeastern Regional Institute of Science and Technology, Nirjuli, India |
| pYUB854 | Recombination sites res1 and res2 flanked by multiple cloning sites, apr^R^ | (1) |
| pMV262 | hsp60 promoter, kan^R^ | (2) |
| pMV262-zeo | hsp60 promoter, zeo^R^ | This study |
|  | | |
| **Primer name** | **Nucleotide sequence** | **Purpose: target gene** |
| gyrA_F1 | GCATCTAAAGCCGCTGAGAACG | Sanger sequencing of *gyrA* and *gyrB* |
| gyrA_F2 | GCGGGCATCTCCAACATCGAGG |  |
| gyrA_R1 | GAGGTTGTTCAGCACCACCTTGG |  |
| gyrA_R2 | GGTCCACGGGGCGTTCGTTTGC |  |
| gyrB_F1 | GGCGTGGTGACGAGTTTAAAG |  |
| gyrB_F2 | GAGATCTTCGAGACCACCACCTA |  |
| gyrB_F3 | GCAAGAGTGCCACCGATATC |  |
| gyrB_R1 | GTAAGTACGACGGCACAACG |  |
| up_SpeI_F | cgc**actagt**GCCGGAGCCCGCCAGCGC | PCR amplification of *rshA* flanking regions (upstream and downstream) |
| up_HindIII_R | gcg**aagctt**tCATGCCCGGCCCTCCGCCAAC |  |
| down_XbaI_F | cgc**tctaga**CTAGCTGTTGGGACGCTTGC |  |
| down_KpnI_R | cgc**ggtacc**GGACATCGCCTGGTCTGCG |  |
| AES_up1_F | GCAGATTACGCGCAGAAAA | Sanger Sequencing (clone sequence verification): AES |
| AES_up2_R | GACGATGGTCAAGGCATATGC |  |
| AES_apr1_F | CTCTGGCGGATGCAGGAAGATC |  |
| AES_apr2_R | CTCGATCAGTCCAAGTGGCCCATC |  |
| AES_res2_F | CTTCACGAGCAGACCTCACTAG |  |
| AES_down1_F | CTTCACGAGCAGACCTCACTAG |  |
| AES_down2_R | AAAGTATATATGAGTAAACTTGGTC |  |
| ΔrshA_up_F | CAATGAGTTGGCGGAGGGC | Sanger Sequencing: verification of *rshA* deletion |
| ΔrshA_down_R | GCAAGCGTCCCAACAGCTAG |  |

**Supplemental Table 1**. List of plasmids and oligonucleotide primers used in this study.

AES: Allelic Exchange Substrate

**Supplemental Figure 1. Comparative growth of wild type parent *M. abscessus* ATCC 19977, Δ*rhsA* and complemented strains in drug-free broth and on solid medium.** **(A)** *In vitro* growth curves of wild type, and isogenic Δ*rhsA* and complemented strains. Growth curves were determined as described previously (3). Mid-log phase precultures (OD_600_ = 0.4 – 0.6) were diluted to OD 0.025 and grown in 7H9 broth in 490 cm² roller bottles (Corning) at 37°C. The OD was monitored at regular intervals using Ultrospec 10 cell density meter (Biochrom, Holliston, MA, USA). The experiment was carried out twice independently with technical duplicates and one representative set of results is shown. **(B)** Growth and colony morphology of these strains on drug-free 7H10 agar. A loop full of bacteria from log phase cultures (OD 0.05) was streaked on drug-free 7H10 agar plates and images were captured after 5 days of incubation at 37°C.


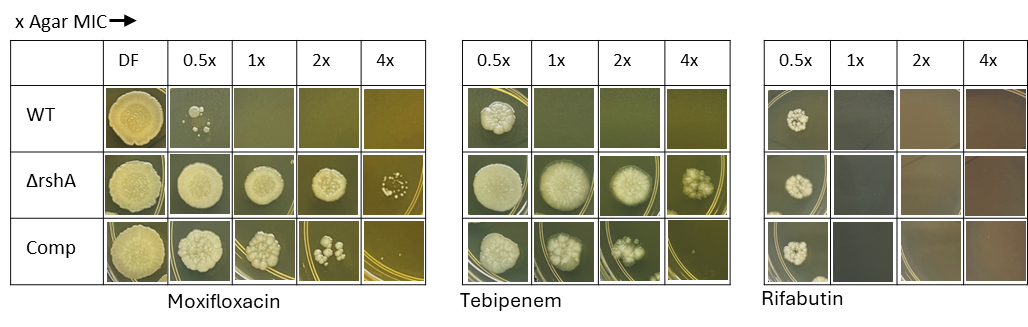


**Supplemental Figure 2**. **Growth inhibition of wild type parent *M. abscessus* ATCC 19977, *ΔrhsA* and complemented strains on solid medium containing multiples of the agar MIC**. Agar MICs were as follows: Moxifloxacin = 100 µM; tebipenem = 100 µM; rifabutin = 12.5 µM. Representative images of bacterial growth are shown for each concentration. The experiment was performed two times independently in technical duplicates, and a representative image is shown. DF: drug-free; WT, wild type ATCC19977; Comp, complemented.

**References**

1. Ganapathy US, Lan T, Krastel P, Lindman M, Zimmerman MD, Ho H, Sarathy JP, Evans JC, Dartois V, Aldrich CC, Dick T. 2021. Blocking Bacterial Naphthohydroquinone Oxidation and ADP-Ribosylation Improves Activity of Rifamycins against Mycobacterium abscessus. Antimicrob Agents Chemother 65:e0097821.

2. Stover CK, de la Cruz VF, Fuerst TR, Burlein JE, Benson LA, Bennett LT, Bansal GP, Young JF, Lee MH, Hatfull GF, et al. 1991. New use of BCG for recombinant vaccines. Nature 351:456-60.

3. Aragaw WW, Cotroneo N, Stokes S, Pucci M, Critchley I, Gengenbacher M, Dick T. 2022. In Vitro Resistance against DNA Gyrase Inhibitor SPR719 in Mycobacterium avium and Mycobacterium abscessus. Microbiol Spectr 10:e0132121.

4. Schildkraut JA, Coolen JPM, Burbaud S, Sangen JJN, Kwint MP, Floto RA, Op den Camp HJM, Te Brake LHM, Wertheim HFL, Neveling K, Hoefsloot W, van Ingen J. 2022. RNA Sequencing Elucidates Drug-Specific Mechanisms of Antibiotic Tolerance and Resistance in Mycobacterium abscessus. Antimicrob Agents Chemother 66:e0150921.
